# Rupture Risk Assessment for Cerebral Aneurysm Using Interpretable Machine Learning on Multidimensional Data

**DOI:** 10.3389/fneur.2020.570181

**Published:** 2020-12-23

**Authors:** Chubin Ou, Jiahui Liu, Yi Qian, Winston Chong, Xin Zhang, Wenchao Liu, Hengxian Su, Nan Zhang, Jianbo Zhang, Chuan-Zhi Duan, Xuying He

**Affiliations:** ^1^National Key Clinical Specialty/Engineering Technology Research Center of Education Ministry of China, Guangdong Provincial Key Laboratory on Brain Function Repair and Regeneration, Department of Neurosurgery, Neurosurgery Institute, Zhujiang Hospital, Southern Medical University, Guangzhou, China; ^2^Department of Biomedical Sciences, Faculty of Medicine and Health Sciences, Macquarie University, Sydney, NSW, Australia; ^3^Monash Medical Centre, Monash University, Clayton, VIC, Australia

**Keywords:** intracranial aneurysm, machine learning, rupture, subarachnoid hemorrhage, stroke

## Abstract

**Background:** Assessment of cerebral aneurysm rupture risk is an important task, but it remains challenging. Recent works applying machine learning to rupture risk evaluation presented positive results. Yet they were based on limited aspects of data, and lack of interpretability may limit their use in clinical setting. We aimed to develop interpretable machine learning models on multidimensional data for aneurysm rupture risk assessment.

**Methods:** Three hundred seventy-four aneurysms were included in the study. Demographic, medical history, lifestyle behaviors, lipid profile, and morphologies were collected for each patient. Prediction models were derived using machine learning methods (support vector machine, artificial neural network, and XGBoost) and conventional logistic regression. The derived models were compared with the PHASES score method. The Shapley Additive Explanations (SHAP) analysis was applied to improve the interpretability of the best machine learning model and reveal the reasoning behind the predictions made by the model.

**Results:** The best machine learning model (XGBoost) achieved an area under the receiver operating characteristic curve of 0.882 [95% confidence interval (CI) = 0.838–0.927], significantly better than the logistic regression model (0.779; 95% CI = 0.729–0.829; *P* = 0.002) and the PHASES score method (0.758; 95% CI = 0.713–0.800; *P* = 0.001). Location, size ratio, and triglyceride level were the three most important features in predicting rupture. Two typical cases were analyzed to demonstrate the interpretability of the model.

**Conclusions:** This study demonstrated the potential of using machine learning for aneurysm rupture risk assessment. Machine learning models performed better than conventional statistical model and the PHASES score method. The SHAP analysis can improve the interpretability of machine learning models and facilitate their use in a clinical setting.

## Introduction

Intracranial aneurysms are present in 3–7% of the population (1). Although the rupture rates of aneurysms are low, the consequences can be dire (2, 3). Surgical or endovascular treatments for aneurysms are effective but still carry the risk of complications (3). Given the high prevalence and catastrophic consequence of rupture, identification of rupture-prone aneurysms is of vital importance.

Morphology and hemodynamics have been shown to be associated with aneurysm rupture (4–9). There are other risk factors such as hypertension (10), blood lipid level (11), alcohol consumption, and smoking (12, 13). Based on these risk factors, various risk evaluation methods have been proposed. The PHASES score is among the most quoted, which is derived based on several large cohort studies (14, 15). In both ISUIA and UCAS studies, aneurysms smaller than 7 mm have been associated with very low risk profiles for rupture (3). However, it was also reported that more than 47% of ruptured aneurysms were of size <5 mm (16). Because of the complex nature of aneurysm rupture estimation, the rupture risk assessment of aneurysms remains a challenging problem.

Machine learning is a group of algorithms that function to train a computer to learn complex nonlinear relationships by observing a large amount of data. There has been growing interest in the use of machine learning to predict aneurysm rupture. Some of these prediction models have been developed based on morphological features (17–19), whereas others have been based on hemodynamic features (20). As the rupture of the aneurysm is clearly secondary to multifactorial causes, the use of only morphological and hemodynamic features may result in missing important information from other risk factors. Moreover, machine learning models are usually more complex as they operate as “black boxes” and therefore difficult to interpret, thereby potentially limiting their use in a clinical setting.

In this study, to address the complex nature of aneurysm rupture, we integrated multiple aspects of information from patient demographics, lifestyle behaviors, clinical histories, lipid profile results, and aneurysm morphology to develop rupture risk models. To conquer the black box problem of machine learning output and improve its interpretability, we further applied the Shapley Additive Explanations (SHAP) method to explain the reasoning behind the prediction made by the model. We aimed to provide a useful tool to aid decision making in the management of cerebral aneurysms.

## Materials and Methods

### Study Population

Approval for this study was obtained from the local institutional review board. The data were anonymous, and the requirement for informed consent was therefore waived. The data in the current study were obtained from 2016 to 2019 from a single center. The inclusion criteria included (1) a diagnosis of aneurysm/s by digital subtraction angiography (DSA) or computed tomography angiography (CTA). The exclusion criteria included (1) fusiform aneurysm; (2) presence of other intracranial vascular malformation; (3) traumatic, bacterial, dissecting, or fusiform aneurysm; (4) cases with poor image quality not adequate for morphology measurement; and (5) cases with missing information in regard to morphology, medical histories, lipid profile results, and lifestyle behaviors. The data that support the findings of this study are available from the corresponding author upon reasonable request.

### Overall Research Plan

Morphological variables, lifestyle variables, laboratory test results, and clinical variables were acquired for each patient. All the variables were first examined by statistical test. Risk models were developed using conventional statistical method and three different machine learning methods, namely, support vector machine (SVM), artificial neural network (ANN), and gradient boosting tree. The four models and the PHASES score method were compared in terms of their predicting performance. Following that, SHAP analysis was applied to the best model to determine the impact of each feature and reveal the reasoning behind the output of the model.

### Data Acquisition

Morphological parameters including aneurysm size, aneurysm height, aneurysm width, neck width, parent artery diameter, aspect ratio, and size ratio were measured and calculated from three-dimensional (3D) DSA images according to their definition in previous research (21). The measurement was performed on 3D volume-rendering images. The operator first identified the location of neck and rotated the view angle such that the maximum length (size) of aneurysm was revealed. The operator then measured morphological parameters mentioned above. For detailed definition of the morphological parameters, see Supplementary Figure 4. The measurements were done by two independent neurosurgeons blinded to the rupture status, and the average of their readings was used. Blood tests were also performed for patients to measure lipid levels. Patient demographic characteristics, medical history, and lifestyle behaviors were also recorded. The list of collected variables is shown in Supplementary Table 1.

### Model Construction

To compare the efficacy of conventional statistical model and machine learning models, logistic regression (LR) and three typical machine learning algorithms were selected to construct the prediction model, which were SVM, ANN, and extreme gradient boosting (XGBoost). LR model has been extensively used in clinical research and is well known for its simplicity and straightforward interpretation. An SVM tries to find optimal decision boundary—hyperplanes that best separate data of different categories. An ANN is a collection of connected nodes (neurons) that compute the output by some nonlinear functions of the sum of its input. During training, the connections (weights) between neurons are modified so that computers can learn the pattern to classify data. XGBoost is an ensemble learning method that constructs multiple decision trees to classify data (22). During the training process, new trees are sequentially added to correct the errors made by existing trees. The final prediction is a weighted sum of all tree predictions.

### Model Training and Evaluation

**Figure 1:**
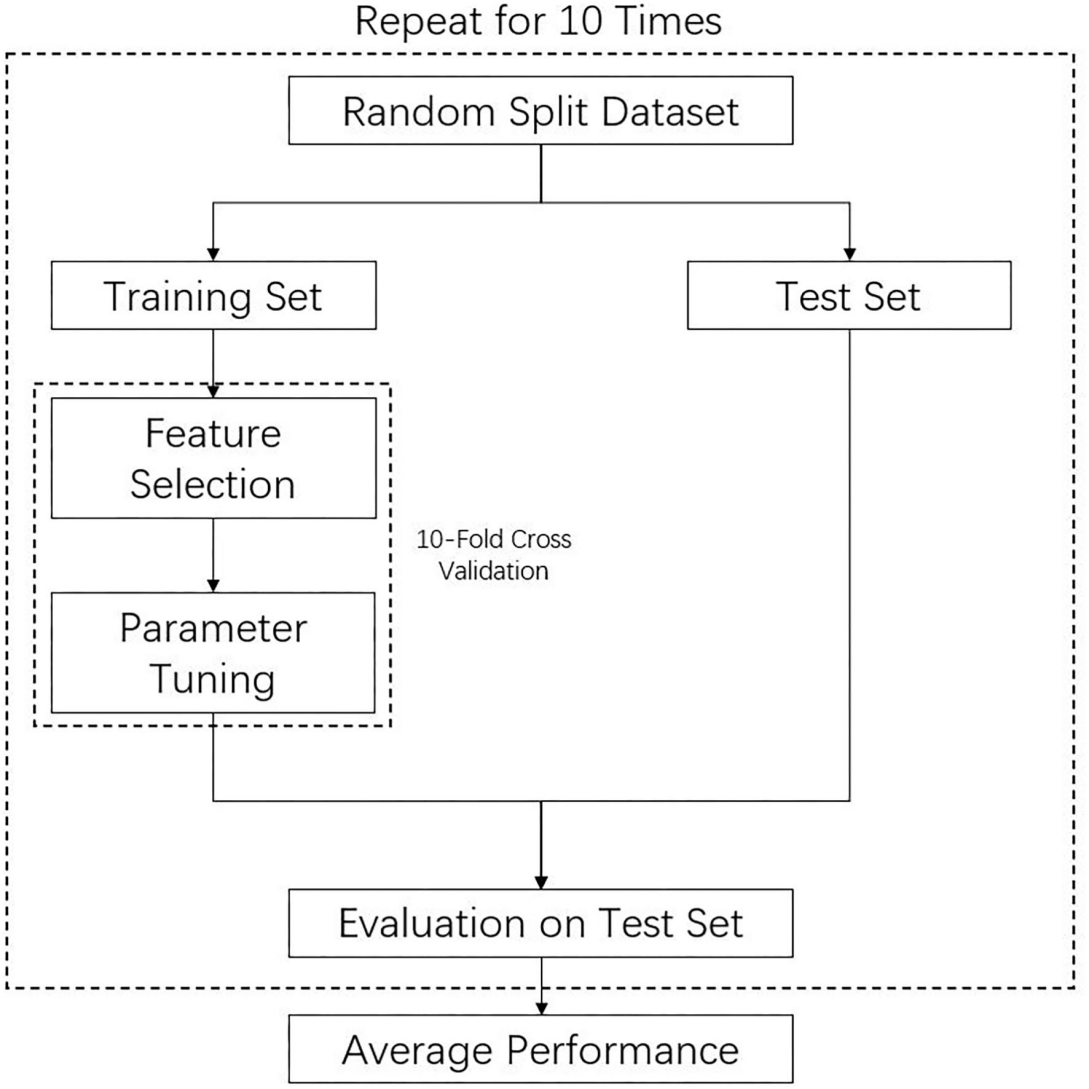
Training and evaluation procedures of machine learning model.

The overall training procedures are shown in Figure 1. The whole data samples were randomly split into training and test sets according to a division of 7:3. Optimal features and hyperparameters combinations for the model were determined on the training set. Tenfold cross-validation (23) was used in the process of feature selection and hyperparameters. Details of the feature selection and hyperparameter tuning were described in Supplementary Material. The model was subsequently tested on the independent test set, which had not been seen by the model during the training process so as to avoid overfitting. To avoid bias due to random split of the training and test sets, the above procedures were repeated for 10 times, and the performance of different models was compared. The comparison of different models' performance in the 10 repeats was examined by Wilcoxon signed ranks test as suggested by a previous study (24). All continuous variables were normalized to the range of 0 to 1. Categorical variables were transformed into binary variables using one-hot encoding. As unruptured aneurysms make up the majority of cases, which may bias the model, we therefore used the balanced accuracy as training metric that is a balanced measure of the quality of a binary classifier for imbalanced class problems (25). Besides commonly used metrics such as area under the receiver operating characteristic (ROC) curve, we also reported results of area under the precision recall curve, which is more informative on imbalanced dataset. The four machine learning models were also compared to the PHASES score method, which was calculated based on aneurysm size, location, and patient clinical information such as hypertension, age, and previous subarachnoid hemorrhage history (14). Scores from different aspects were added up together, and higher score indicates higher risk. For example, a score of 4 corresponds to a 5-year rupture risk of 0.9%, whereas a score of 20 corresponds to risk of 17.8%.

### Model Interpretation

Machine learning models are often seen as black boxes. However, for clinical decision making, the reasoning behind the diagnosis is very important. Therefore, it is important to understand what features lead to algorithm output. To conquer the black box nature of machine learning method, we applied the SHAP (26) method to the best model obtained above to interpret the predictions made by the model. The SHAP method has been developed from cooperative game theory, and it serves to calculate the contributions of each feature value toward the final prediction. The above machine learning models and SHAP analysis were implemented using Scikit-Learn library (27) (https://scikit-learn.org/stable/) and SHAP (https://github.com/slundberg/shap) in Python.

### Statistical Analysis

All features between ruptured and unruptured cases were compared using univariate analyses. For binary or categorical features, Fisher exact test or χ^2^ test was performed. For continuous features, they were first examined with the Shapiro-Wilk test to determine normality, followed by the Student *t*-test (for normally distributed variables) or Mann-Whitney *U* test (for non–normally distributed variables). *P* < 0.05 was considered to be statistically significant. After that, variables with *P* < 0.05 were further selected into further analysis. These variables were also tested for collinearity using the Pearson test. Only linearly independent variables (*P* > 0.05, correlation < 0.8) were input into multivariate analysis. Backward conditional stepwise method was used to derive the LR model. Statistical analyses were performed using SPSS (IBM Corporation, USA). The comparison of ROC curves was based on the method of DeLong et al. (28) using MedCalc (MedCalc Software, Belgium). Comparisons between multiple groups were corrected by Bonferroni correction.

## Results

A total of 390 patients and 452 aneurysms were recruited, 374 of which were included in the current study. The baseline statistics of both ruptured and unruptured groups are presented in Table 1. For demographic variables, gender and hypertension were significantly different between the two groups. For lifestyle behaviors variables, smoking, alcohol consumption, and intensive physical activity were significant variables. In terms of aneurysm morphology, aneurysm size, vessel angle, size ratio, aspect ratio, location, shape, and multiplicity were all significantly different between the two groups. For blood test variables, triglyceride level and homocysteine were significant variables.

**Table 1:** Results of univariate analysis for all feature variables.

**Parameters**	**Unruptured (*n* = 306)**	**Ruptured (*n* = 68)**	***P***
Age, y	56.16 ± 11.17	52.85 ± 11.62	0.154
Female	208 (68.3%)	31 (45.6%)	0.001^*^
BMI, kg/m^2^	22.69 ± 3.16	22.55 ± 3.18	0.160
Hypertension			0.001^*^
No	223 (59.0%)	36 (42.4%)	
Grade I	67 (18.8%)	21 (21.2%)	
Grade II	13 (3.3%)	6 (6.1%)	
Grade III	3 (0.3%)	5 (6.1%)	
Smoking			0.020^*^
Yes	39 (12.7%)	17 (25.0%)	
Alcohol			0.032^*^
Yes	32 (10.4%)	14 (20.6%)	
Physical activity			0.023^*^
Moderate–heavy	83 (27.1%)	29 (42.6%)	
Sleep			0.608
Lack of sleep	155 (50.6%)	46 (67.6%)	
Hyperlipidemia			0.952
Yes	13 (4.2%)	3 (4.4%)	
Previous SAH			0.186
Yes	80 (26.1%)	23 (33.8%)	
Diabetes			0.266
Yes	21 (6.9%)	7 (10.3%)	
TG	1.48 ± 1.47	1.16 ± 0.61	0.001^*^
Cholesterol	4.50 ± 0.93	4.57 ± 1.23	0.677
LDL	2.69 ± 0.87	2.75 ± 0.99	0.545
HDL	1.30 ± 0.32	1.26 ± 0.51	0.942
Hcy	10.65 ± 4.19	12.09 ± 4.83	0.005^*^
Aneurysm size	3.63 ± 1.51	4.33 ± 1.56	0.005^*^
Aneurysm width	3.62 ± 1.60	4.03 ± 1.85	0.152
Aneurysm height	3.28 ± 1.38	3.52 ± 1.43	0.139
Aneurysm neck	3.53 ± 1.28	3.25 ± 1.51	0.070
Vessel angle	99.72 ± 28.24	110.08 ± 30.05	0.006^*^
Size ratio	1.33 ± 0.76	2.12 ± 1.14	<0.001^*^
Aspect ratio	1.00 ± 0.50	1.20 ± 0.50	0.002^*^
Multiplicity			<0.001^*^
Yes	120 (39.2%)	10 (14.7%)	
Location			<0.001^*^
ICA	196 (64.1%)	12 (17.6%)	
MCA	39 (12.7%)	16 (23.5%)	
ACA	13 (4.2%)	4 (5.9%)	
PCA	5 (1.6%)	1 (1.5%)	
BA	11 (3.6%)	3 (4.4%)	
VA	6 (1.9%)	2 (2.9%)	
AComA	15 (4.9%)	8 (11.8%)	
PComA	21 (6.9%)	22 (32.3%)	
Aneurysm shape			<0.001^*^
Regular	271 (88.5%)	42 (61.7%)	
Daughter sac	5 (1.6%)	13 (19.1%)	
Multilobulated	9 (2.9%)	4 (5.9%)	
Others	21 (6.9%)	9 (13.2%)	

**Figure 2:**
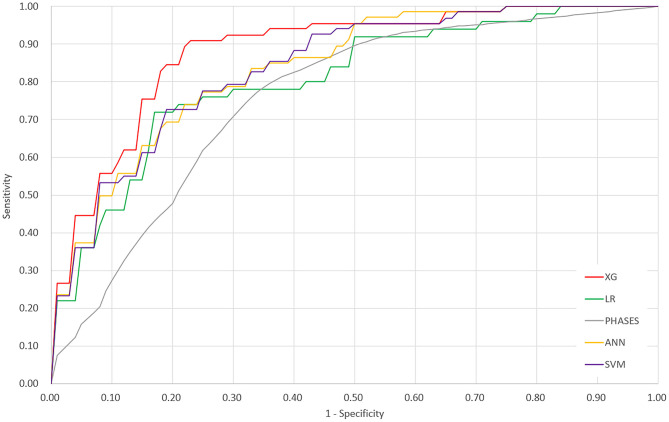
Receiver operating characteristic curves of the four derived models and the PHASES score method. XG, XGBoost; ANN, artificial neural network; SVM, support vector machine; LR, logistic regression.

The ROC curves of the four derived models and the PHASES score method are plotted in Figure 2. The XGBoost model achieved the highest area under the ROC curve of 0.882 [95% confidence interval (CI), 0.838–0.927], followed by the SVM model of 0.838 (95% CI = 0.790–0.886), ANN model of 0.837 (95% CI = 0.794–0.881), and LR model of 0.779 (95% CI = 0.729–0.829). The PHASES score method achieved an area under the ROC curve of 0.757 (95% CI = 0.713–0.800). SVM and ANN models performed better than LR, but the difference did not reach statistical significance. The XGBoost model performed significantly better than LR model (*P* = 0.002) and PHASES score method (*P* = 0.001). Table 2 summarizes the performances of all the models.

**Table 2:** Performance comparison of machine learning models, logistic regression model, and the PHASES score method.

	**XGBoost**	**ANN**	**SVM**	**LR**	**PHASES**
Area under the ROC curve	0.881	0.837	0.838	0.801	0.758
Sensitivity	90.9%	74.0%	72.6%	72.0%	79.7%
Specificity	77.0%	78.0%	81.0%	83.0%	64.0%
Balanced accuracy	0.839	0.760	0.765	0.775	0.718

**Figure 3:**
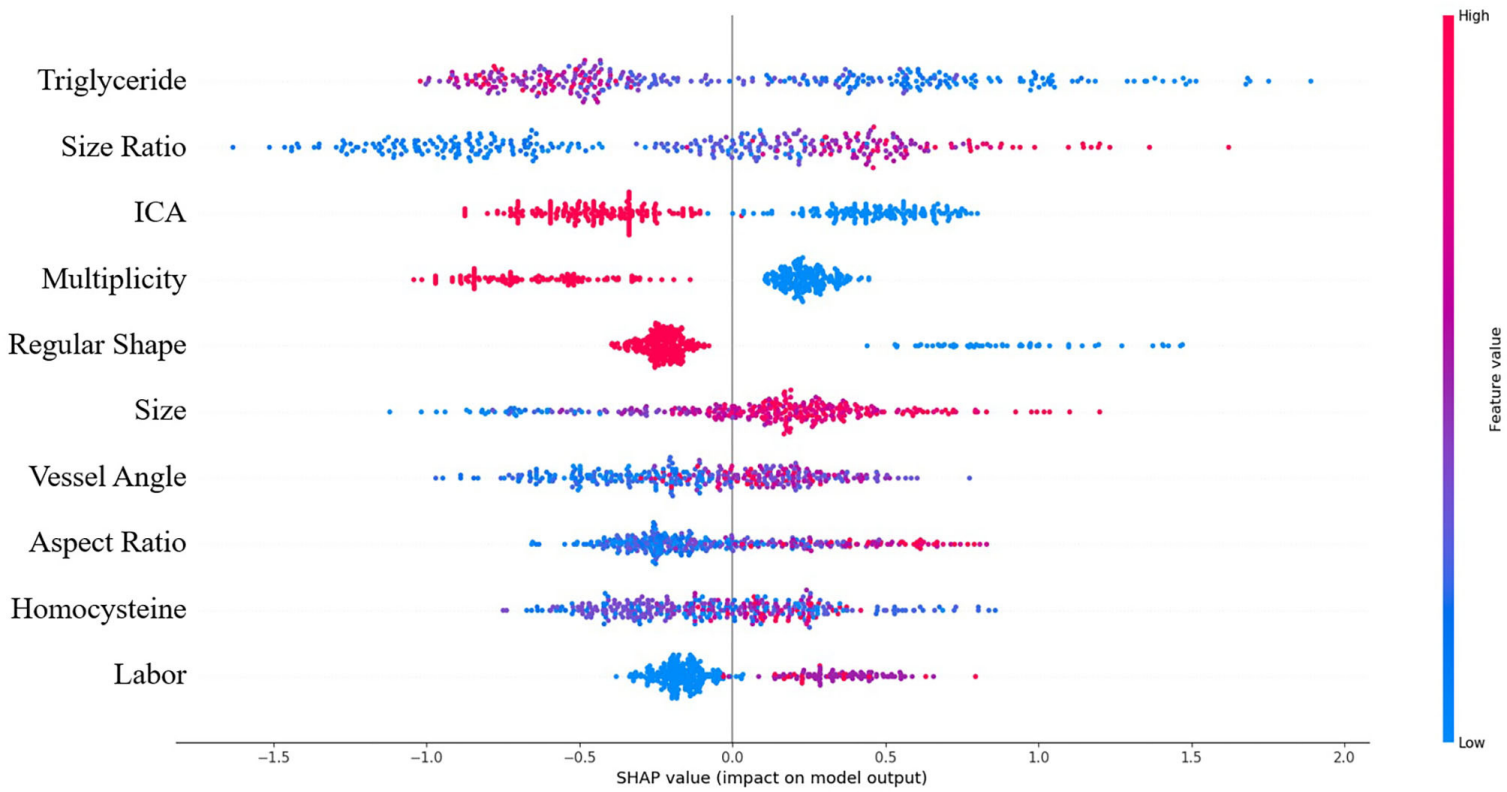
Summary of SHAP analysis on the dataset. This shows the 10 most important features and their impact on the model output. Each dot represents a case in the dataset. The color of a dot indicates the value of the feature, with blue meaning the lowest range and red meaning the highest range. The horizontal axis shows the corresponding SHAP value of the feature. A positive SHAP value contributes to the prediction of rupture and vice versa.

SHAP analysis revealed the relative importance of each feature in the XGBoost model. Location at internal carotid artery (ICA), size ratio, and triglyceride level were the three most important features, as shown in Figure 3. The model tended to associate large size ratio, lower triglyceride level, larger vessel angle, larger aspect ratio, and intensive occupational physical activity with positive SHAP values, which means increased risk. On the contrary, location at ICA, regular shape, and multiple aneurysms were associated with negative SHAP values, which means decreased risk.

**Figure 4:**
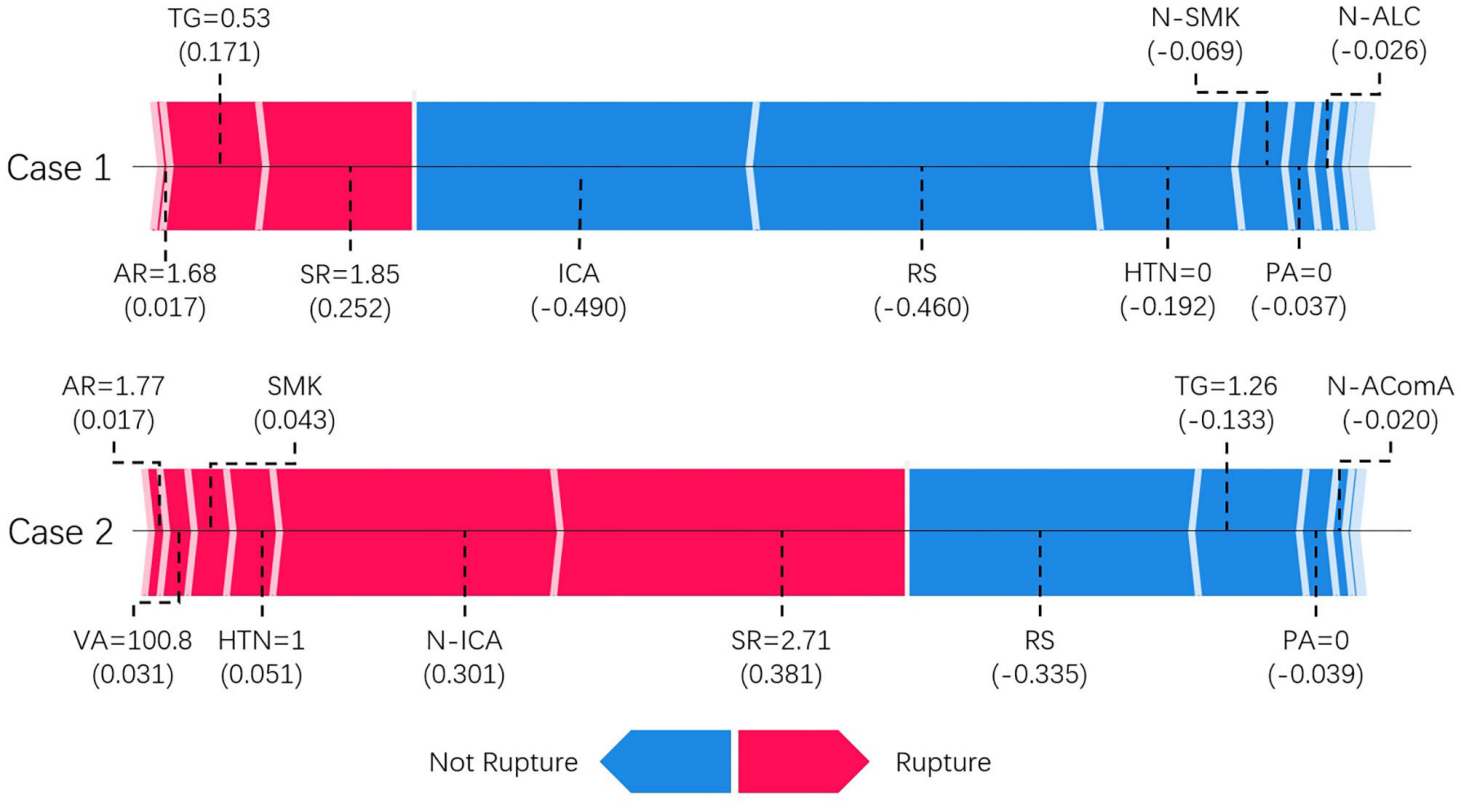
SHAP model explanation of two typical predictions. This shows the main contributing features behind the model prediction. Features linked to red color bar contribute to rupture prediction, whereas features linked to blue color bar contribute to unruptured prediction. The length of the color bar represents the amount of contribution, measured by SHAP value shown in parenthesis, from the corresponding feature. TG, triglyceride; SR, size ratio; RS, regular shape; AR, aspect ratio; NW, neck width; HTN, hypertension; PA, physical activity; VA, vessel angle; N-ICA, aneurysms not on internal carotid artery; N-AComA, aneurysms not on anterior communicating artery.

Figure 4 shows two typical predictions made by the XGBoost model. SHAP analysis revealed the contribution from each input feature toward the model output, thus revealing the underlying reasoning for the prediction. Features that increase the risk of rupture are colored in red and appear on the left-hand side. In contrast, features that decrease the risk of rupture are colored in blue and appear on the right-hand side. The length of the stripe for each feature denotes the importance (weight) of that feature in making the prediction. A longer stripe indicates that the feature contributes more toward or opposes the prediction. If the total length of red stripes is longer than that of blue stripes, which means rupture-prone factors outweigh rupture-protected factors, the model will favor the prediction of rupture and vice versa. For example, the first case is an ICA aneurysm correctly classified as unruptured. Being located on ICA, having regular shape and absence of hypertension are the main reasons for unruptured prediction, outweighing other rupture-prone factors such as slightly high size ratio and aspect ratio. The second case is an middle cerebral artery (MCA) aneurysm correctly classified as a ruptured aneurysm. Large size ratio, being located on non-ICA, presence of hypertension, and smoking are the main reasons behind for this case. Therefore, despite some rupture-protected factors such as having regular shape and normal level of triglyceride, it is predicted to be a rupture-prone aneurysm.

## Discussion

We have demonstrated the feasibility of using machine learning to develop aneurysm rupture risk models using multi-aspects data obtained from patient demographics, clinical characteristics, lifestyle behaviors, lipid profiles, and angiographic images. The best model (XGBoost) showed good performance with area under the ROC curve of 0.882, better than the model derived using LR and the PHASES score method. We further demonstrated that by using the SHAP method the reasoning behind the model prediction can be revealed.

Size is an important risk factor in rupture as confirmed by the ISUIA study and UCAS study (2, 3). Location is also an important factor to consider. Aneurysms located on the anterior and posterior communicating arteries are known to bear an increased risk of rupture, while aneurysms located on the internal carotid arteries seldom rupture. Morphological parameters such as size ratio, aspect ratio, and daughter sac have also been associated with rupture (4, 5, 17, 21). Our model has learned similar patterns. The SHAP analysis showed that ICA, size ratio, aspect ratio, and vessel angle had significant impact on the model risk output. It should be noted that all non-ICA aneurysms were associated with positive SHAP values (increased risk), meaning increased risk for aneurysms at other locations, consistent with previous studies (29).

Our study also included four lifestyle behaviors variables. In statistical analysis, smoking and alcohol consumption were associated with increased risk, which is consistent with previous findings (12, 13). We further discovered that intensive occupational physical activity was associated with increased risk, which correlates with the findings from two studies (30). However, as the sample size of current study is relatively small, studies with more cases and multicenter design should be performed to further investigate the association.

Hyperlipidemia and lipid accumulation have been suggested to be related to aneurysm rupture (18, 31–33). Triglyceride is commonly recognized as a risk factor for cardiovascular disease. However, it is interesting to note that in our study triglyceride levels exhibited the opposite trend. We observed that triglyceride level was significantly lower in the ruptured group (*P* = 0.001). This pattern was also recognized by the algorithm; therefore in the model, a low level of triglyceride was conceived as a risk factor. The association between triglyceride and cerebral aneurysm rupture has been seldom discussed in the literature. There are some studies reporting increased risk of hemorrhagic stroke associated with a low triglyceride level (34–38). Although the mechanism is not fully understood, it has been suggested that low cholesterol may lead to necrosis of smooth muscle cells in arterial medial layer (39), therefore making the arterial wall more susceptible to rupture.

In the current study, machine learning models performed better than conventional statistical model such as LR. Although machine learning models are powerful, they are often more complex, which makes them difficult to understand like a “black box.” This is a significant drawback if machine learning models were to be used in clinical setting. Clear reasoning is very important in medical decision making, especially for deadly disease such as cerebral aneurysms. We demonstrated that by using the SHAP method, machine learning models can be made more interpretable, and the underneath reasoning behind each prediction can be revealed, which can facilitate its use in clinical setting.

Extensive efforts have been made to stratify the risk of aneurysm rupture. Most of the previous research surrounding evaluation of aneurysm rupture risk has developed their models on conventional regression methods (4, 5, 8, 14). Although such models are simple and robust, they are limited to the use of a relatively small number of features and assume linear relationships between each feature and the risk of rupture. Machine learning allows for the development of a more flexible relationship between feature and risk, with more features involved in the final calculation. Liu et al. developed an ANN for the rupture prediction of AComA aneurysms (17). Their model was developed mainly based on morphological parameters and achieved an area under the ROC curve of 0.928. Moreover, Liu et al. developed a prediction model using Lasso regression based on radiomics features derived from angiographic images (18) and achieved area under the ROC curve of 0.853. Kim et al. applied deep convolutional neural network to classify the rupture risk of small aneurysms based on angiographic images and achieved an area under the ROC curve of 0.755 (19). Silva et al. also developed a random forest model and achieved an area under the ROC curve of 0.81 (40). The rupture of cerebral aneurysm is inherently a multifactorial consequence. Therefore, in the current study, we applied a more holistic approach by taking account of information from morphologies, demographics, medical history, lifestyle behaviors, and lipid profile. Furthermore, to make our model more interpretable, we applied the SHAP method to reveal the underlying reasoning behind predictions made by the machine learning model.

### Clinical Application

Our model has demonstrated good performance and improved interpretability. Although in the current study, the measurement of morphological parameters was done on DSA images, it can be generalized to CTA and magnetic resonance angiography images. The morphology measurement can be done on site and does not require upload of full set of images, which minimizes the risk of sensitive information leakage. The input variables required by the model can be easily obtained, and the calculation time was only of seconds, thus making the prediction model easily applicable to real-world clinical environment. The improved interpretability revealed the reasoning behind the algorithm output, which could give more confidence to users. In the future, we plan to make it as a cloud-based service, on which users can input required variables and receive feedback of risk analysis in real time, thus more accessible to the public.

There are several limitations in our study. The major limitation is the retrospective nature of the study. Unruptured aneurysms at diagnosis did not guarantee no rupture in the long term. The follow-up period in our study is short (mean follow-up time: 59 days), but our model can still help to identify high-risk aneurysms that need immediate treatment. We considered only a limited number of morphology parameters. Although we have considered more than a dozen of factors in our study, some risk factors, such as sophisticated morphological parameters and computational hemodynamics and use of statin, were not included in the current study. Further study incorporating these factors is needed. The number of ruptured and unruptured cases in our study is not well balanced, which may affect the generalization of the developed machine learning model. The number of cases from a single center is relatively small, and the model has not been validated externally. Multicenter prospective study with long-term follow-up will be needed to further validate the model.

## Conclusion

We have demonstrated the feasibility of evaluating aneurysm rupture risk using model derived from machine learning algorithm based on multidimensional data of morphologies, demographics, clinical characteristics, lifestyle behaviors, and lipid profiles. The developed model showed promising performance with good interpretability, with potential to further optimize the management of unruptured aneurysms.

## Data Availability Statement

The raw data supporting the conclusions of this article will be made available by the authors upon reasonable request.

## Ethics Statement

The studies involving human participants were reviewed and approved by Institutional Review Board of Zhujiang Hospital. Written informed consent from participants was not required in accordance with institutional requirements and local legislation.

## Author Contributions

C-ZD and CO: conceptualization. JL, XZ, WL, HS, NZ, JZ, XH, and C-ZD: data curation. JL: data preprocessing. CO: computer programming. CO and JL: data analysis. CO, YQ, and WC: manuscript drafting. CO, JL, YQ, WC, XZ, WL, HS, NZ, JZ, XH, and C-ZD: manuscript editing. All authors contributed to the article and approved the submitted version.

## Conflict of Interest

The authors declare that the research was conducted in the absence of any commercial or financial relationships that could be construed as a potential conflict of interest.
