# Supplementary material for: Rupture Risk Assessment for Cerebral Aneurysm Using Interpretable Machine Learning on Multidimensional Data
Source: Front Neurol. 2020 Dec 23;11:570181. doi: 10.3389/fneur.2020.570181 (PMC7785850; doi:10.3389/fneur.2020.570181)
Supplement: Supplementary file 1 [file Table_3.DOCX]

Supplemental Material

**Supplemental Methods**

Supplemental Table 1 List of Collected Parameters

| Demographics |  |
| --- | --- |
| Age | Gender |
| Body Mass Index |  |
| Clinical Histories |  |
| Hypertension (4 Categories: No, Grade I, Grade II, Grade III) | Hyperlipidemia |
| Previous Subarachnoid Hemorrhage | Diabetes |
| Morphology |  |
| Aneurysm Size | Neck Width |
| Vessel Angle | Aneurysm Height |
| Aneurysm Width | Parent Artery Diameter |
| Size Ratio | Aspect Ratio |
| Multiplicity | Shape (4 Categories: Regular, Daughter Sac, Multilobulated, Other Type) |
| Location (8 Categories: ICA, MCA, ACA, PCA, BA, VA, AComA, PComA) |  |
| Lipid Profile |  |
| Total Cholesterol | Triglyceride |
| LDL | HDL |
| Lifestyle Behaviors |  |
| Smoking (3 Categories: No, Mild, Heavy) | Alcohol Consumption (3 Categories: No, Mild, Heavy) |
| Sleeping (4 Categories: <6 hrs, 6-7 hrs, 7-8 hrs, 8-9 hrs, >9hrs) | Occupational Physical Activity (3 Categories: Mild, Moderate, Heavy, Very Heavy) |

Supplemental Table 2 Result of Multivariate Analysis

|  | Odds Ratio | P |
| --- | --- | --- |
| Gender | 0.508 (0.240-1.074) | 0.076 |
| TG | 0.276 (0.130-0.584) | 0.001* |
| Aneurysm Size | 0.799 (0.628-1.016) | 0.067 |
| Neck Width | 0.745 (0.547-1.015) | 0.062 |
| Size Ratio | 2.533 (1.627-3.946) | <.001* |
| Multiplicity | 0.243 (0.102-0.584) | 0.002* |
| Location | 1.241 (1.102-1.397) | <.001* |
| Shape | 1.526 (1.020-2.285) | 0.040* |

Ten-fold Cross-Validation

In the current study, feature selection and hyperparameter tuning were conducted on the training set. For a fixed validation set with few cases, this can easily introduce overfitting. To avoid overfitting, we used ten-fold stratified cross-validation. The general procedure in our study was as follow: (a) randomly split the training set into 10 folds, with each fold having approximately equal ratio of ruptured and unruptured cases; (b) take each fold as the validation fold in each repeat; (c) take the remaining nine fold in that repeat to be the derivation fold (sometimes also termed training fold); (d) train model with a specific set of features or hyperparameters and evaluate the model on the validation fold; (e) get the evaluation score. The (a) to (e) procedures were repeated for ten times and the average score from the ten repeats were recorded.


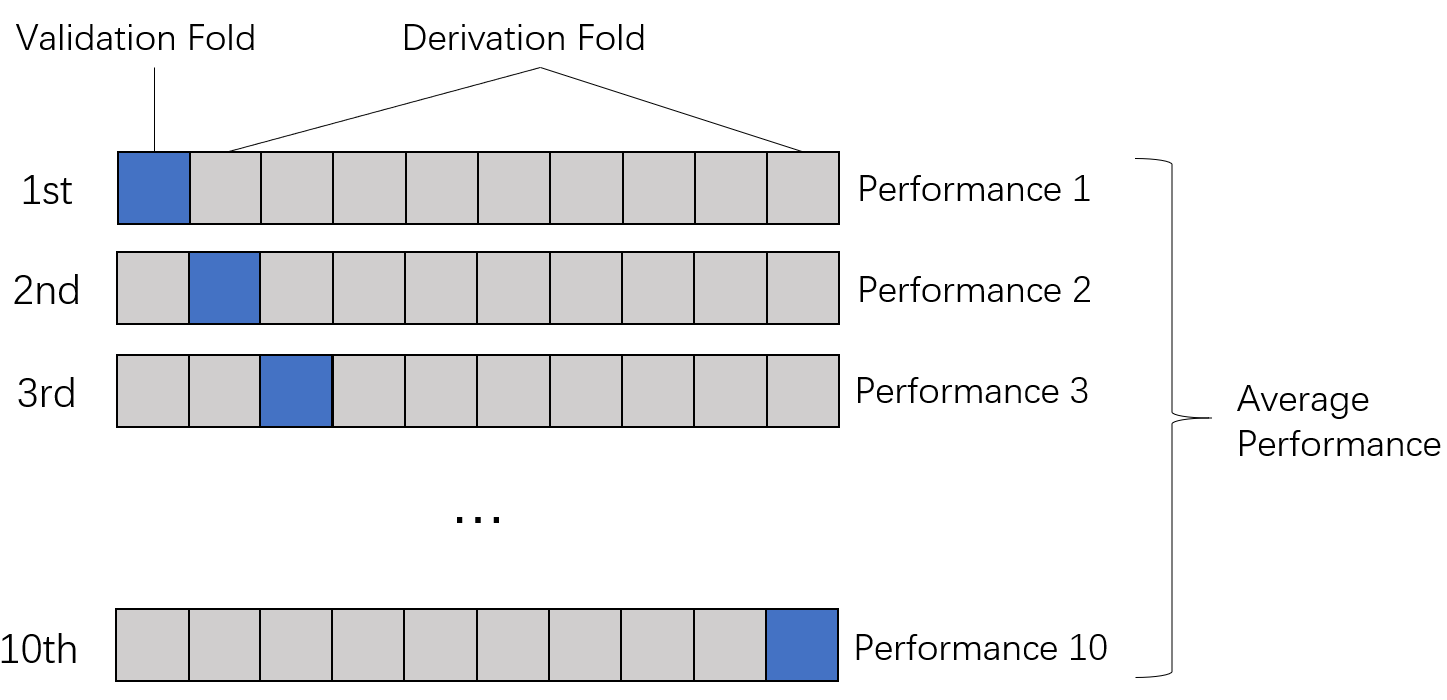


Supplemental Figure 1 Illustration of ten-fold cross-validation process

Feature Selection

The general procedures were as follow: (a) a random forest classifier was initially trained with a full set of features on the derivation set and evaluated on the validation set; (b) the least important feature (lowest information gain) was removed from the model recursively; (c) after each removal of feature, the classifier was retrained and reevaluated on the validation set. Steps (a) to (c) were repeated ten times using the ten-fold cross-validation. In a typical run, the average performances of the ten validations against different number of features were plotted in Supplementary Figure 2. The optimal numbers and combination of features were obtained when the classifier reached its maximum balanced accuracy on the validation set. The selected features were presented in Supplementary Figure 3 based on their information gain (feature importance).


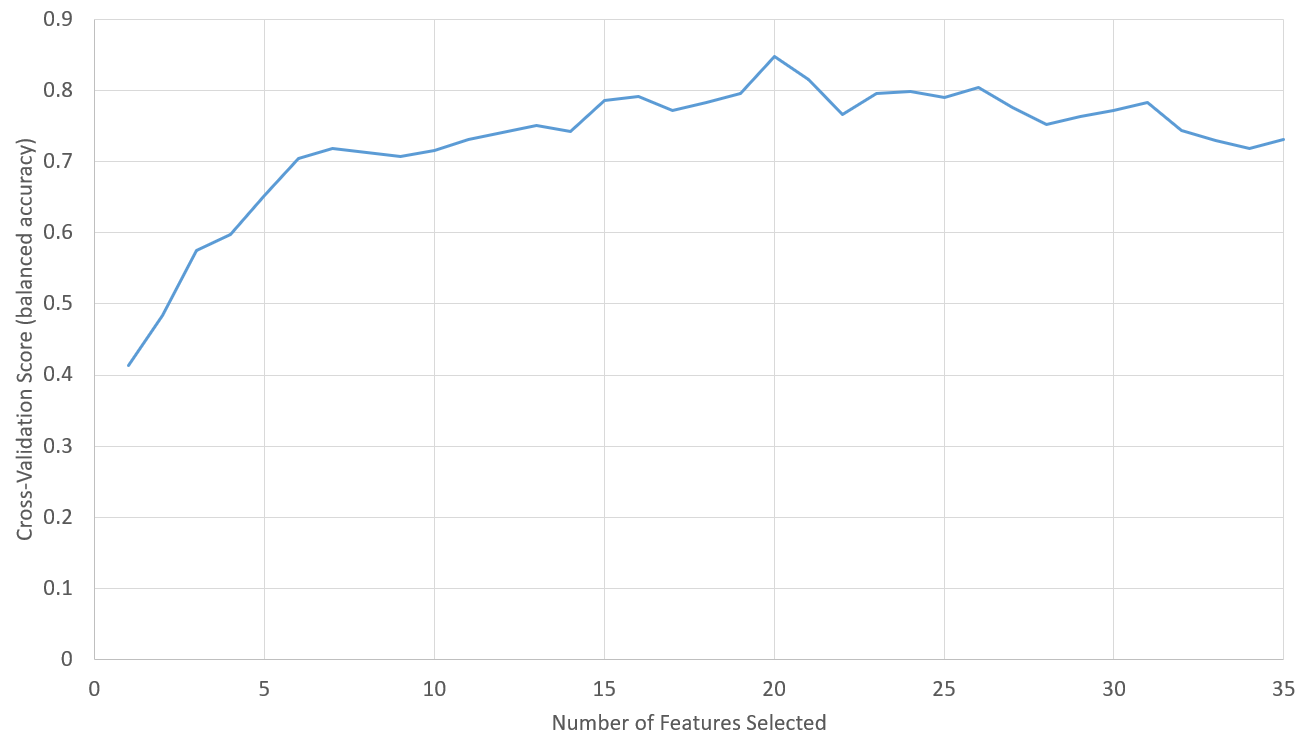


Supplemental Figure 2 Recursive feature elimination. The horizontal axis shows the number of features selected. The vertical axis shows the average performance of the random forest classifier in the ten-fold cross-validation. The optimal number of features was determined to be 20 when the balanced accuracy was at maximum.


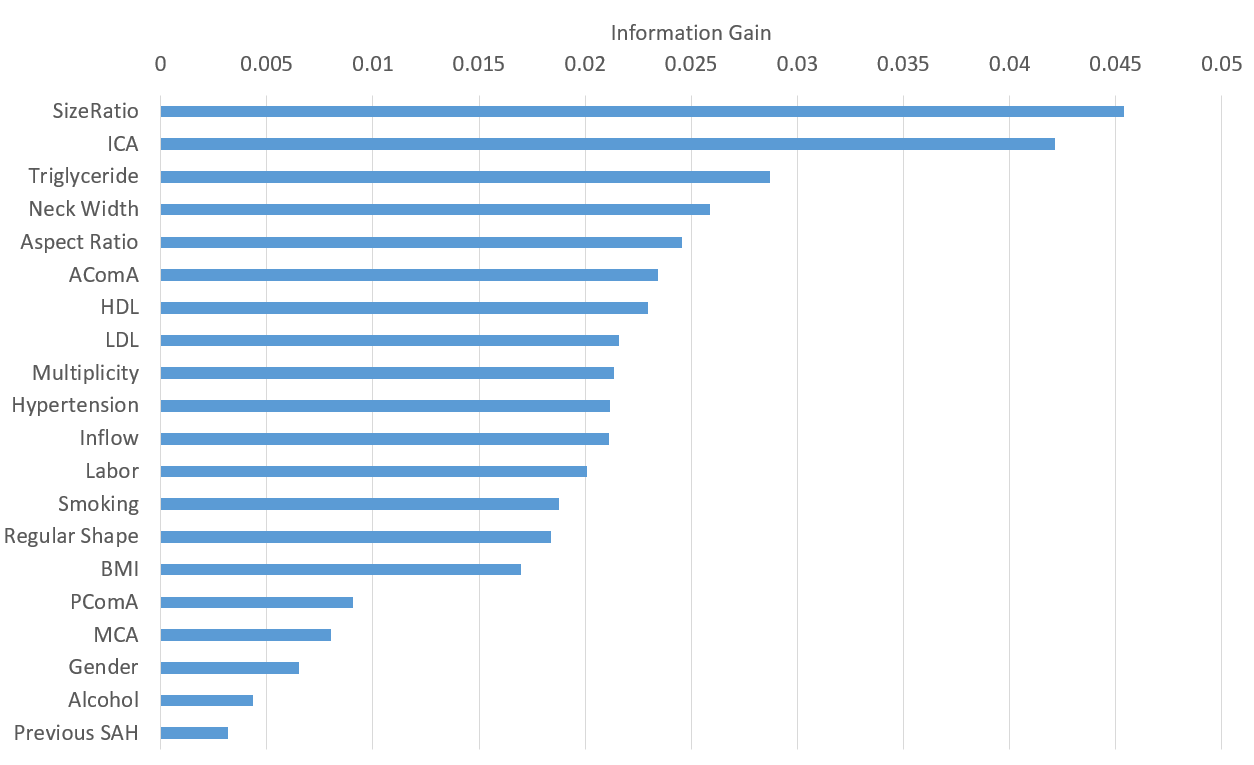


Supplemental Figure 3 Features selected and their information gain ranking in a typical run. (Labor: Occupational Physical Activity; Inflow: Vessel Angle; BMI: Body-mass Index; Previous SAH: Previous Subarachnoid Hemorrhage)

Hyperparameter Tuning

Hyperparameters were tuned using ten-fold cross-validation on the derivation set. Randomized grid-search was used to search for optimal hyperparameters. The hyperparameters tuned for each model were listed in Supplemental Table 3.

|  | Hyperparameters |
| --- | --- |
| Logistic Regression | default |
| Support Vector Machine | kernel type, C, degree class_weight |
| Artificial Neural Network | hidden_layer_sizes, reg_alpha, learning rate, activation, max_iter |
| XGBoost | n_estimators, max_depth, learning rate, gamma, min_child_weight, subsample, colsample, reg_alpha, reg_lambda |

Aneurysm Morphology Measurement


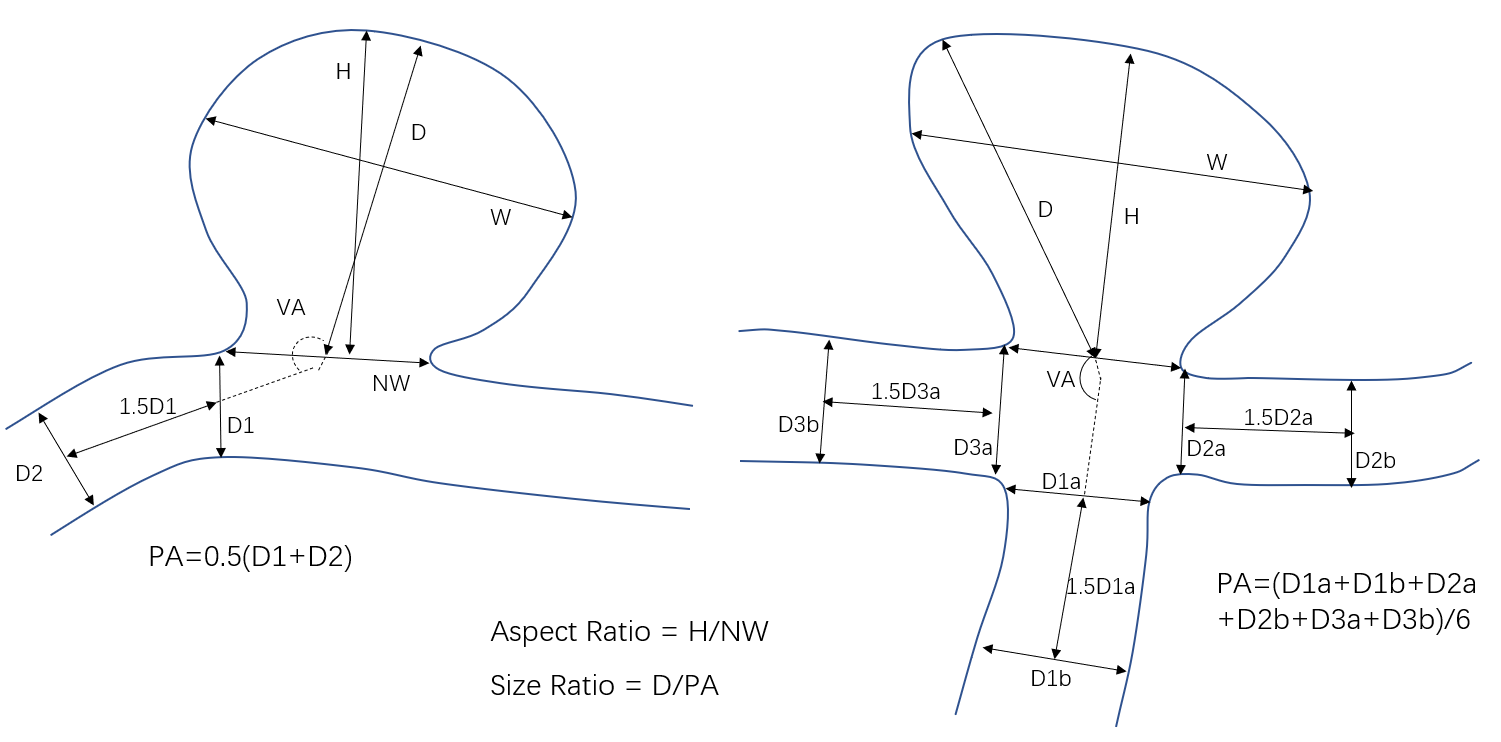


Supplemental Figure 4 Schematics of aneurysm morphology measurement. D=Aneurysm Size, H=Aneurysm Height, W=Aneurysm Width, NW=Neck Width, VA=Vessel Angle, PA=Parent Artery Diameter.

Supplemental Table 4. Predictors, criteria, and points for the PHASES score calculation

| Aspect | Criteria | Score |
| --- | --- | --- |
| Population | North American / European (except Finnish) | 0 |
|  | Japanese | 3 |
|  | Finnish | 5 |
| Hypertension | Yes | 1 |
|  | No | 0 |
| Age | Less than 70nyears | 0 |
|  | 70 or older | 1 |
| Aneurysm Size | <7mm | 0 |
|  | 7-9.9mm | 3 |
|  | 10-19.9mm | 6 |
|  | >=20mm | 10 |
| Earlier SAH | Yes | 1 |
|  | No | 0 |
| Location | ICA | 0 |
|  | MCA | 2 |
|  | ACA, Posterior Circulation, PComA | 4 |
